# Improvement effects of a novel Chinese herbal formula in imiquimod and IL-23-stimulated mouse models of psoriasis

**DOI:** 10.1186/s13020-024-00951-9

**Published:** 2024-06-11

**Authors:** Lan Wang, Yao-Xing Dou, Qiu-Xia Yu, Zhen Hu, Siu-Po Ip, Yan-Fang Xian, Zhi-Xiu Lin

**Affiliations:** 1 School of Chinese Medicine, Faculty of Medicine, The Chinese University of Hong Kong, Room 101-J, 1/F, Li Wai Chun Building, Shatin , Hong Kong SAR, NT China; 2 The Second Affiliated Hospital of Guangzhou University of Chinese Medicine, Guangzhou University of Chinese Medicine, Guangzhou, China; 3 Hong Kong Institute of Integrative Medicine, The Chinese University of Hong Kong, Hong Kong SAR, China

**Keywords:** Psoriasis, Inflammation skin disease formula, Imiquimod, IL-23/Th17 axis, MAPK pathway, Jak/Stat pathway

## Abstract

**Background:**

Psoriasis is a long-term inflammatory skin disease. A novel herbal formula containing nine Chinese herbal medicines, named Inflammation Skin Disease Formula (ISDF), has been prescribed in clinics for decades.

**Aims:**

To investigate the efficacy and action mechanisms of ISDF on psoriasis using imiquimod (IMQ) and Interleukin-23 (IL-23)-induced models in mice and reveal the pharmacokinetics profile of ISDF in rats.

**Methods:**

Topical administration of IMQ and intradermal injection with IL-23 respectively induced skin lesions like psoriasis on the dorsal area of Balb/c and C57 mice. The mice's body weight, skin thickness, and psoriasis area and severity index (PASI) were assessed weekly. SD rats were used in the pharmacokinetics study and the contents of berberine and baicalin were determined.

**Results:**

The PASI scores and epidermal thickness of mice were markedly decreased after ISDF treatment in both models. ISDF treatment significantly decreased the contents of IL-17A and IL-22 in the serum of IMQ- and IL-23-treated mice. Importantly, ISDF markedly downregulated IL-4, IL-6, IL-1β, and tumor necrosis factor α (TNF-α) gene expression, and the phosphorylation of NF-κB p65, JNK, ERKs and MAPK p38 in IMQ-treated mice. The protein phosphorylation of Jak1, Jak2, Tyk2 and Stat3 was significantly mitigated in the ISDF-treated groups. The absorption of baicalin and berberine of ISDF through the gastrointestinal tract of rats was limited, and their distribution and metabolism in rats were also very slow, which suggested ISDF could be used in the long-term application.

**Conclusions:**

ISDF has a strong anti-psoriatic therapeutic effect on mouse models induced with psoriasis through IMQ and IL-23, which is achieved by inhibiting the activation of the Jak/Stat3-activated IL-23/Th17 axis and the downstream NF-κB signalling and MAPK signalling pathways. ISDF holds great potential to be a therapy for psoriasis and should be further developed for this purpose.

## Introduction

Psoriasis is characterized by skin lesions of silvery white scales, red plaques and a positive Auspitz’s sign [1]. Around 125 million patients with psoriasis worldwide in 2022 [2]. Histopathologic changes include abnormal epidermal differentiation, inflammatory infiltration, and blood vessel dilation [3]. In addition to skin lesions, psoriasis patients usually have multiple comorbid illnesses such as cardiovascular ailments, psoriatic arthritis, metabolic syndrome, and inflammatory bowel disease [4]. Additionally, they are more likely to experience mental health problems like anxiety and depression [5]. The development of psoriasis is affected by gene and environment [6]. Up to date, psoriasis is believed to be predominantly involved with Th17 cells, which are IL-17-producing cells [7, 8]. Extrinsic and intrinsic stimuli such as smoke, medication, microorganisms, and stress could lead to the over-release of pro-inflammatory cytokines from epidermal keratinocytes. Furthermore, dendritic cells present antigens and produce cytokines, stimulating native CD4 + T cells to differentiate into various types (Th1, Th2, Th17, and Tregs). Stimulated Th17 cells induced by IL-23 could lead to an imbalance between these T cells and Tregs, and finally cause skin inflammation and keratinocyte proliferation [9]. In turn, activated keratinocytes subsequently stimulate Th17 cells to secret IL-17A/F, IL-22, and TNF-α, forming an inflammation loop [10].

In general, mild to moderate psoriasis patients are usually treated with oral glucocorticoids (e.g. dexamethasone) or topical steroids, but their side effects cannot be ignored, such as causing acne-like rash, skin atrophy, telangiectasia, pigmentation, hormone-dependent dermatitis and so on [11]. For patients with severe psoriasis, systemic biologics like ustekinumab (mAb against interleukins), TNF-α and IL-17 inhibitors like secukinumab and ixekizumab have been successfully applied in patients with excellent efficacy, but the high costs prevent the popularity of biologics in most patients [12]. Many psoriasis patients opt for Chinese herbal medicine as a safer and less expensive alternative to Western medicine.

According to the theory of Chinese medicine, psoriasis is called “Bai Bi” (White Boil in Chinese) in Chinese medicine based on its unique features of the psoriatic plaques. Pathologically, psoriasis is thought to be caused by blood heat, blood stasis, and skin dryness in the chronic stage. In Chinese medicine treatment, heat-clearing and blood-cooling Chinese herbs such as *Moutan Cortex*, *Rhinoceros Unicornis*, *Rehmanniae Radix*, *Phellodendri Chinensis Cortex*, as well as wind-expelling and itchiness-stopping herbs like *Dictamni Cortex* are commonly prescribed in clinical treatment of psoriasis. The potent efficacy and clinical safety of Chinese herbal medicines in psoriasis management have attracted the attention of physicians and researchers. In recent years, we have developed a Chinese herbal medicine formula named Inflammation Skin Disease Formula (ISDF), for treating several inflammatory diseases. ISDF is a proved prescription made by Prof. Zhi-Xiu Lin, who is a registered doctor of Chinese medicine in Hong Kong [13]. ISDF contains 9 herbs such as the dried root of *Rehmannia glutinosa* Libosch (*Rehmanniae Radix*), the dried root of *Paeonia lactiflora* Pall (*Paeoniae Radix Rubra*), the dried root of *Scutellaria baicalensis* Georgi (*Scutellariae Radix*), the dried cortex of *Phellodendron chinense* Schneid. (*Phellodendri Cortex*), the dried fruit of *Forsythia suspensa* (Thunb.) Vahl. (*Forsythiae Fructus*), the dried seed of *Plantago asiatica* L. (*Plantaginis Semen*), the dried seed of *Vigna umbellata* (Thunb.) Ohwi et Ohashi. (*Vignae Semen*), the dried root barks of *Dictamnus dasycarpus* Turcz. (*Dictamni Cortex*) and the dried ripe seed of *Kochia scoparia* (L.) Schrad. (*Kochiae Fructus*). Previously, we reported that ISDF was effective in relieving atopic dermatitis (AD)-like symptoms in dinitrochlorobenzene (DNCB)-induced in vivo model. Its anti-inflammatory and anti-allergic activities contributed to the anti-AD efficacy. Therefore, we hypothesized that ISDF also possesses an anti-psoriatic effect in animal models of psoriasis.

To test the hypothesis, we initially assessed the anti-psoriatic effectiveness of ISDF in preclinical psoriasis models. The animal models for psoriasis research include spontaneously produced models (Ab mice, flaky tail mice), trans-genetically produced models (HLA-B27 transgenic rats; mice with IFN-γ/IL-17A over-expression in epidermal), xenotransplantation produced models (human tissue onto mice), T-cell transfer produced models (CD4^+^/CD45RB^hi^ T-cell transfer) and directly induced models (sensitization with IMQ/IL-23) [14]. In this study, two drug-induced psoriasis models were adopted, as the IMQ-induced and IL-23-induced models. The IMQ-induced model is the most common because of its easy accessibility, low cost, and convenience [15]. And the IL-23-induced model is established by intradermally injecting the recombinant mouse IL-23 antibody into the skin [16]. This model is a better representation of human psoriasis pathology compared to the IMQ-induced model, which has not been reported as a drug screening model before, probably because the high price of the recombinant IL-23 restricts its widespread application [16]. This study used these two models to evaluate the anti-psoriasis effect of ISDF in vivo and also aimed to unravel its therapeutic target focusing on the IL-23/Th17 axis.

## Materials and methods

### Authentication of raw herbal materials of ISDF

The crude decoction pieces of ISDF were purchased from Guangzhou Zisun Pharmaceutical Company. Each of the component herbs was authenticated following the methods described in the Chinese Pharmacopoeia 2020.

### Extraction and preparation of ISDF

Briefly, the raw herbs were ground and mixed, followed by extraction with 60% ethanol and ultrasonic extraction as described in our previous study [13]. The total extracts were finally vacuum dried and stored at −20 ℃. The ISDF was found to contain 4.92% of baicalin, 2.90% of berberine, 0.26% of paeoniflorin and 0.10% of phillyrin as reported in our previous study [13].

### Animals

Adult female Balb/c or C57 mice and male Sprague–Dawley rats were provided by Laboratory Animal Services Center, CUHK, and used for the establishment of the IMQ or IL-23 mouse model and pharmacokinetics study, respectively. The animals were kept in the animal holding room where in a light/dark cycle, with temperature under 23 °C, and humidity at 50%, and had free access to a standard diet and distilled water. All procedures were approved by the Animal Experimentation Ethics Committee at CUHK (Reference no.: 21/125/ITF).

### Inducement with IMQ and ISDF treatment

**Fig. 1:**
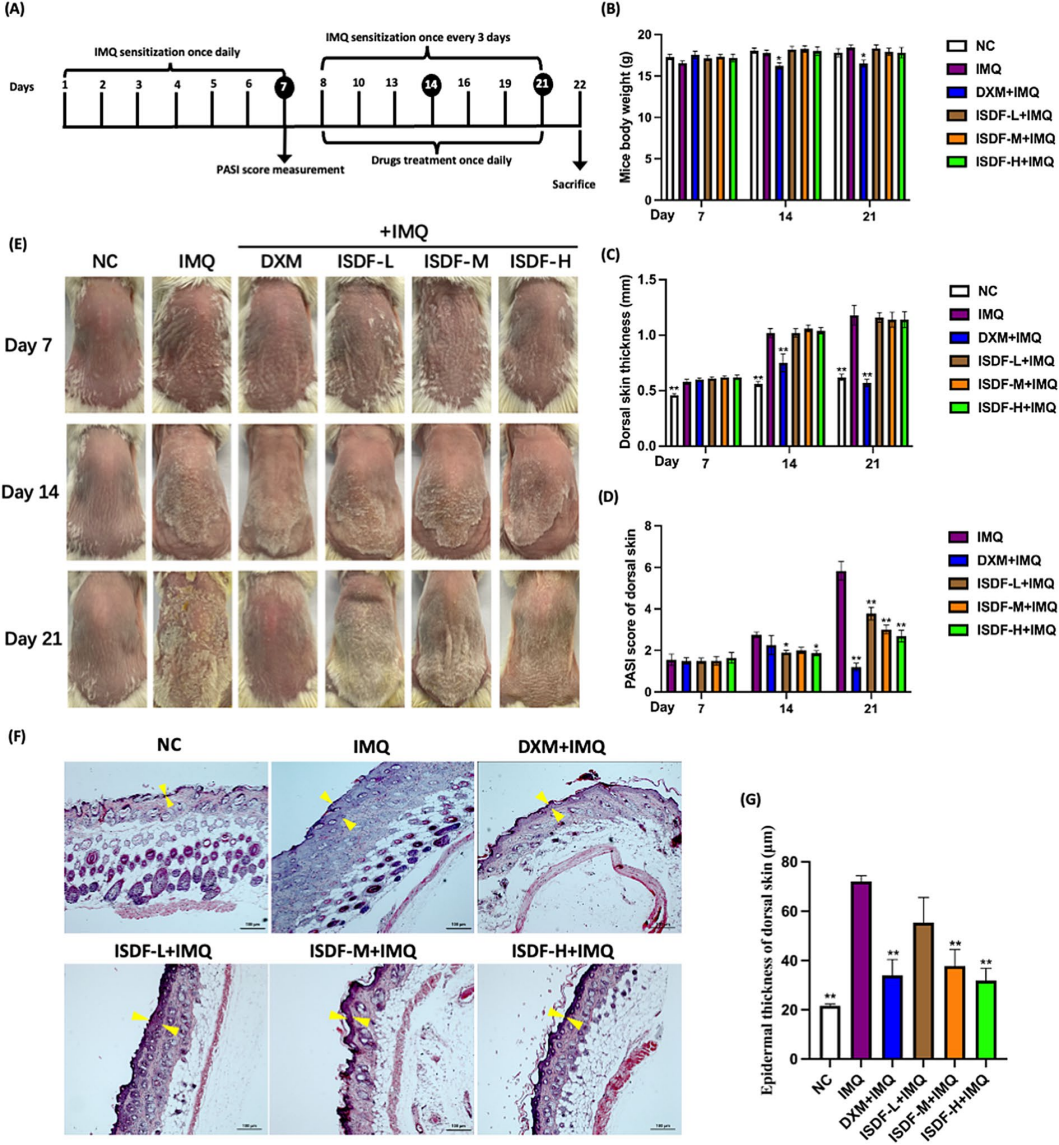
Effects of ISDF on IMQ-induced mouse psoriasis model. **A** The procedure of IMQ inducement and ISDF treatment in mice. **B** Body weight. **C** Dorsal skin thickness. **D** PASI score. **E** Represent photos. **F** H&E-stained photos. Magnification at 100 × . **G** Epidermal thickness (indicated by arrow). Data were presented as mean ± SEM (n = 8). *p < 0.05 and **p < 0.01 compared with the IMQ group

A total of 48 Balb/c mice were randomly assigned to: (a) normal control group (NC); (b) IMQ group; (c) IMQ plus dexamethasone (DXM, as the positive control, 3 mg/kg) group; and (d-f) IMQ plus ISDF (3.15, 6.30 and 9.45 g/kg) groups. All mice, except the NC group, were topically dosed with 62.5 mg Aldara (iNova pharmaceuticals, Australia) daily on the shaved dorsal skin. After the seven-day sensitization of IMQ, the psoriasis symptoms appeared on the dorsal area. Between day 8 and day 22, Aldara cream was applied once every three days for a total of six applications, with each application consisting of 62.5 mg of the cream being applied to the same area. ISDF and DXM were suspended in 0.5% CMC-Na and orally given to the mice once daily for two weeks. Treatments were initiated seven days after the first IMQ sensitization. The mice in the NC group and the IMQ group were administered with only the vehicle for two weeks. (Fig. 1A).

### Inducement with IL-23 and ISDF treatment

**Fig. 2:**
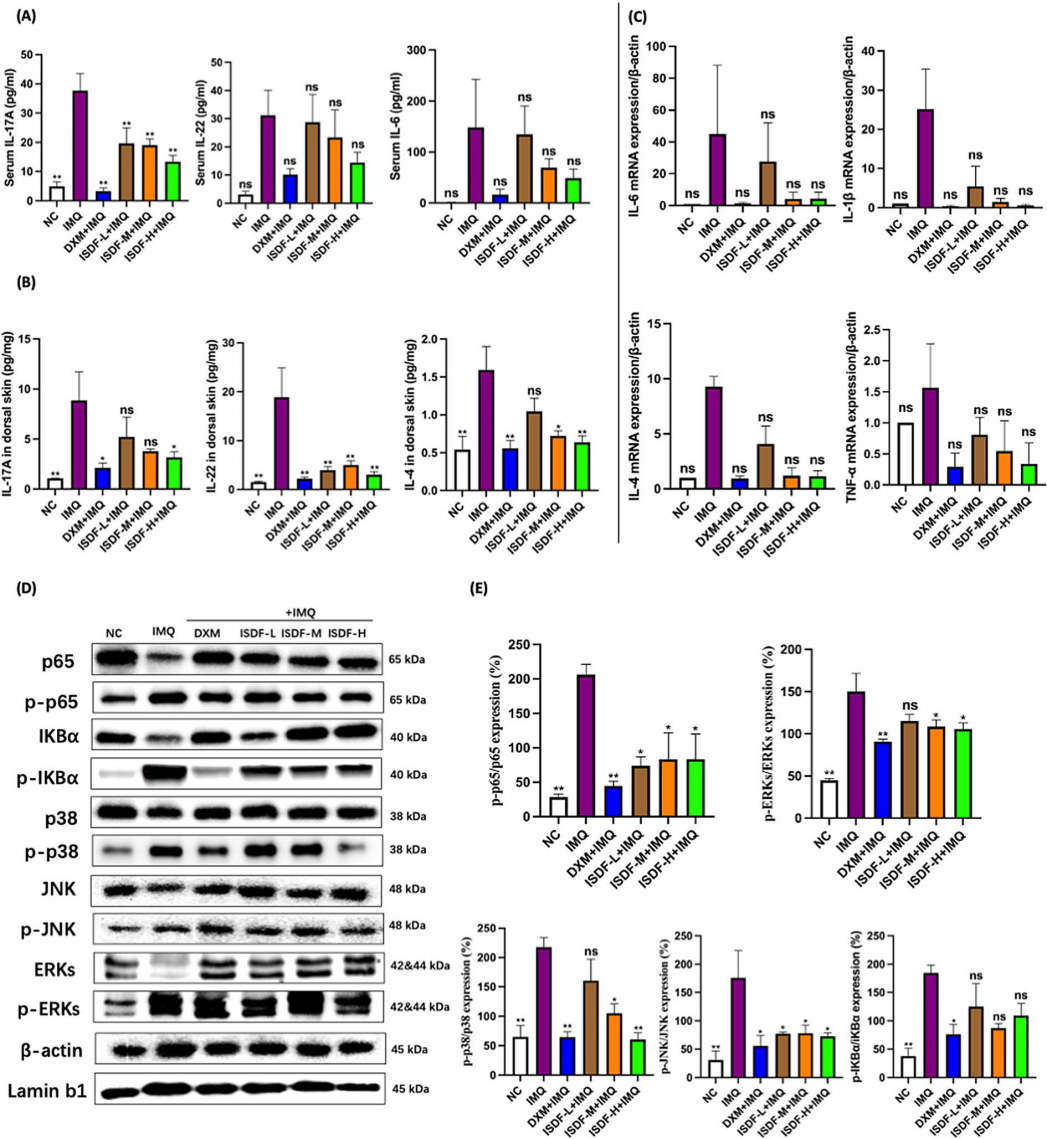
Effects of ISDF on inflammatory response induced by IMQ in mice. **A** Serum levels of IL-17A, IL-22 and IL-6. **B** Skin levels of IL-17A, IL-22 and IL-4. **C** Gene expression of IL-4, IL-6, IL-1β and TNF. **D, E** Protein expression of NF-κB and MAPK pathways. **D** Western blotting bands. **E** Results of quantitative analysis. Data were presented as mean ± SEM (n = 8). *p < 0.05 and **p < 0.01 compared with the IMQ group

A total of 48 C57 mice were randomly assigned to: (a) NC group; (b) IL-23 group; (c) IL-23 plus DXM (3 mg/kg) group; and (d–f) IL-23 plus ISDF (3.15, 6.30 and 9.45 g/kg) groups. Firstly, mice were pre-treated with ISDF or DXM before IL-23 injection. 0.5% CMC-Na was used to suspend ISDF and DXM which were orally given to mice once daily for two weeks, and the mice in the NC group and the IL-23 group were treated with only the vehicle for two weeks. The IL-23 administration was initiated 7 days post the first ISDF/DXM treatment. From day 8, mice concurrently orally received with ISDF/DXM treatment and intradermal injections of recombinant mouse IL-23 (Biolegend, USA) in two locations on the shaved dorsal skin at 1 μg/mouse/day using a 30-gauge needle for seven days. Sterile saline was used as the vehicle control for the NC group. After the seven-day sensitization of IL-23, the psoriasis symptoms appeared on the dorsal area of the mice [15]. The weight of the mice and their dorsal skin thickness was monitored (on day 1, 7, and 14). On the last day, the scoring of the skin psoriasis severity was determined according to PASI after the last drug treatment (Fig. 2A).

### Evaluation of the psoriatic symptoms

The severity of psoriatic lesions on the dorsal area of mice was assessed by psoriasis area and severity index (PASI). The researcher scored the degree of erythema and skin scaling on a scale of zero to four, where 0: no symptoms, 1: slight, 2: moderate, 3: severe, and 4: very severe [17]. The dorsal skin thickness was measured using an electronic vernier caliper which was placed on the middle line of the dorsal area.

### Biological analysis of the inflammatory cytokines in mice

After the last drug treatment, blood samples were harvested and stored at room temperature for one hour, and then centrifuged at 3500 rpm for 20 min. The ELISA kits were used to collect the supernatant and determine the contents of IL-17A (ab199081), IL-22 (ab223857), IL-6 (ab222503) (Abcam, USA) and IL-23 (BMS6017, Thermo Fisher Scientific, USA). After being sacrificed, mouse skin tissues were collected, homogenized with cell extract buffer from Abcam, USA, and centrifuged at 12,000 rpm for 30 min (at 4 °C). The skin samples were analyzed for total protein contents using a BSA assay kit. The concentrations of IL-4, IL-17A, IL-22 and IL-23 in the supernatant were determined.

### Histopathological examination

The dorsal skin tissues were harvested and fixed in 4% paraformaldehyde (PFA) for one day and then embedded in paraffin, followed by section, deparaffinization and rehydration. Five-micrometre sections of dorsal skin were stained with hematoxylin & eosin and observed through a light microscope (Zeiss AxioObserver Z1, Germany) to assess histopathological changes.

### Real-time PCR analysis on the dorsal skin tissues

The same experimental procedure was used as described in our previous study [13]. In the present study, we conducted Mouse TaqMan gene expression assays using the following assay IDs: IL-4 (Mm00445259_m1), IL-6 (Mm00446190_m1), IL-1β (Mm00434228_m1), TNF-α (Mm00443258_m1) and β-actin (Mm02619580_g1).

#### Western blot analysis

The experimental procedure was described in our previous study [13]. The primary antibodies (p65 (#8242, 3033), IKBα (#4812, 2859), JNK (#67,096, 4668), ERKs (#4695, 4370), p38 (#8690, 4511), Jak1 (#3344, 74,129), Jak2 (#3230, 3776), Tyk2 (#35,615, 68,790), Stat3 (#8768, 9145), β-actin (#8457), and Lamin b1 (#13,435)) were purchased from CST, USA.

#### Evaluation of pharmacokinetics profile of ISDF in rats

The rats were split into two groups, with 6 animals each: (a) an untreated control group, and (b) a group that received ISDF at a dosage of 18.90 g/kg. The rats were fasted overnight but had access to distilled water. Blood samples, around 0.4 mL, were harvested from the orbital bleeding while they were under anaesthesia with isoflurane. The blood samples were collected at several time points (5, 15, 30, 60, 120, 240, 480, and 1440 min) after the oral administration of ISDF to the rats. The blood was then centrifuged at 5000 rpm for 20 min (at 4 °C) to separate the plasma (upper) and blood cells (lower).

The plasma (0.2 mL) was added with 1 M/L KH_2_PO_4_ (0.2 mL) and 1 mL methanol and acetonitrile (1:1) and vortexed for 10 min to precipitate protein, and then centrifugated at 10,000 rpm for five min to collect the supernatant. The supernatant was filtered, dried under nitrogen and then re-dissolved in methanol (0.2 mL) and stored at 4 °C [18].

The blood cells were washed thrice with cold 0.9% saline, and then centrifuged at 3500 rpm for five min. The resulting erythrocytes were suspended in cold 0.9% saline (0.5 mL). Afterwards, the erythrocytes were mixed with an equal volume of 15% hydrochloric acid and heated at 60 ℃ for 6 h. They were then centrifugated at 12,000 rpm for 10 min to collect the supernatant, which was filtered and dried under nitrogen. Finally, it was re-dissolved with 0.2 mL methanol [19].

The HPLC method was used to identify and quantify baicalin in plasma and berberine in erythrocytes, as previously published [20].

#### Statistical analysis

All the data were shown as mean ± SEM. One-way ANOVA followed by the Bonferroni test was performed using GraphPad Prism v9.0. Statistical significance between experimental groups was considered when *p* < 0.05. The pharmacokinetics profile of ISDF was calculated using Drug and Statistics (DAS, China) software v2.0.

## Results

### Anti-psoriasis effect of ISDF in IMQ-induced mice model

As depicted in Fig. 1B, the body weight of mice did not show any significant change across all groups on day 7 of the IMQ application. However, on days 14 and 21, DXM application led to a remarkable decrease in mice body weight (*p* < 0.05 *v.s.* the NC group). This indicates that DXM can have notable side effects on the body weight. However, there was no obvious body weight loss in the IMQ-treated group or ISDF-treated groups when compared to the NC group, indicating that the application of IMQ alone or ISDF did not cause significant side effects on the body weight.

Figure 1C shows that all IMQ-treated groups showed increased dorsal skin thickness after one week of application of IMQ (*p* < 0.05 *v.s.* the NC group). After the two-week treatment, the dorsal skin thickness significantly reduced by 51.69% in mice treated with DXM (*p* < 0.01 *v.s.* the IMQ group). However, the increased thickness of the dorsal skin was not affected after two weeks of ISDF treatment. It remained at the same level as that of the IMQ group on day 21. This suggests that ISDF treatment was not effective in reducing the skin thickening induced by IMQ.

### Evaluation of the severity of psoriasis symptoms of IMQ-treated mice

As shown in Fig. 1D, skin scaling and erythema induced by IMQ were found in the IMQ-treated mice on days 14 and 21. However, after two weeks of treatment with ISDF or DXM, the area of dorsal skin with scaling was markedly reduced, especially in ISDF (at the dose of 9.45 g/kg)-treated mice and DXM-treated mice. The recovery effects of ISDF at the low and middle doses could also be observed but were inferior to the high dose of ISDF and DXM. Based on the PASI score evaluated on day 21 (Fig. 1E), the scores in ISDF and DXM-treated mice significantly attenuated when compared with the score of the IMQ group (*p* < 0.01 for all). The groups treated with ISDF at doses of 3.15, 6.30, and 9.45 g/kg and DXM showed significant decreases in PASI scores of 35.16%, 48.54%, 54.03% and 79.41%, respectively (*v.s.* the IMQ group). ISDF treatment reduced the PASI score in a dose-dependent manner, demonstrating the effectiveness of ISDF in ameliorating psoriatic lesions.

### Evaluation of the epidermal thickness of the mice induced by IMQ

Remarkable epidermal thickening and dense infiltration of inflammatory cytokines were observed in the epidermis and dermis of mice induced by IMQ (Fig. 1F, G). The IMQ group showed a significant increase of 2.33 times (*p* < 0.01 *v.s.* the NC group) in the epidermal thickness. However, in DXM-treated and ISDF-treated mice (at a dose of 6.30 and 9.45 g/kg), the epidermal thickness decreased significantly by 52.75%, 47.49%, and 55.62%, respectively (all *p* < 0.01 *v.s.* the IMQ group). The inhibitory effect of ISDF (at 3.15 g/kg) on IMQ-induced epidermal thickening in mice was found to be only marginal. However, the results suggest that ISDF can effectively and dose-dependently suppress the thickening of the epidermal thickness induced by IMQ.

### Evaluation of the inflammatory cytokines of the mice induced by IMQ

An over-release of serum IL-17A is a characteristic feature of psoriasis. As can be seen in Fig. 2A, repeated applications of IMQ significantly stimulated the release of serum IL-17A (by 6.61 times *v.s.* the NC group) in the IMQ group. The serum IL-17A in mice treated with ISDF at doses of 3.15, 6.30, and 9.45 g/kg and DXM significantly decreased by 47.86%, 49.37%, 64.61% and 91.29% respectively (all *p* < 0.01 *v.s.* the IMQ group). In the groups treated with ISDF, the contents of serum IL-22 and IL-6 showed a tendency to decrease, without statistically significance compared with the IMQ group (Fig. 2A). Additionally, the ISDF-M group, the ISDF-H group and the DXM group showed a significant reduction in the contents of serum IL-22 by 25.17%, 53.91%, and 67.47%, respectively. Similarly, the level of serum IL-6 was also significantly reduced by 53.43%, 67.12% and 88.91%, respectively, when in a comparison with the IMQ group. However, the efficacy of ISDF at the dose of 3.15 g/kg on the IMQ-induced increasing release of IL-22 and IL-6 was only marginal. These results suggested that 14 day treatment with ISDF (at 6.30 and 9.45 g/kg) could markedly attenuate the concentrated inflammatory cytokines in the serum of mice induced by IMQ.

In Fig. 2B, it is demonstrated that repeated application of IMQ significantly stimulated the release of IL-17A (6.61 times), IL-22 (11.42 times) and IL-4 (1.94 times) in the dorsal skin of mice in the IMQ-treated group (*v.s.* the NC group). DXM and ISDF (at a dose of 9.45 g/kg) effectively reduced the content of IL-17A by 75.98% and 64.26% (*p* < 0.05 *v.s.* the IMQ group). The inhibitory effects of ISDF were only slightly noticeable when administered at doses of 3.15 and 6.30 g/kg. However, when ISDF was given at the dose of 9.45 g/kg, it showed a significant decrease in the contents of IL-22 (by 84.01%) and IL-4 (by 60.37%) when compared with the IMQ group. The anti-inflammatory effect of ISDF administered at 9.45 g/kg was similar to that of DXM, highlighting its strong anti-inflammatory properties.

Repeated IMQ application could upregulate the gene expression of IL-4, IL-6, IL-1β and TNF-α in the skin of the IMQ group (Fig. 2C). The gene expression levels of IL-4, IL-6, IL-1β and TNF-α were up-regulated by 8.29-fold, 43.83-fold, 24.18-fold, and 56.84%, respectively, as compared to the NC group. However, DXM and ISDF (at the dose of 9.45 g/kg) could substantially down-regulate the gene expression of L-4, IL-6, IL-1β and TNF-α. Although the gene expression between the ISDF-H group and the IMQ group was not significantly different, the ISDF-H group showed a significant reduction in mRNA expression levels of L-4, IL-6, IL-1β and TNF-α by 87.72%, 90.27%, 97.89%, and 78.20% compared with the IMQ group. The effect of ISDF was dose-dependent and comparable to DXM when administered at 9.45 g/kg.

### Evaluation of the effect of ISDF on NF-κB p65 and MAPK pathways in mice induced by IMQ

As demonstrated in Fig. 2D, E, repeated application of IMQ vastly up-regulated the ratios of p-p65/p65 by 6.26 times, p-IKBα/IKBα by 3.9 times, p-p38/p38 by 2.35 times, p-ERKs/ERKs by 2.33 times and p-JNK/JNK by 4.71 times in the IMQ-treated mice compared to the NC group. After the two-week administration with ISDF at 6.30 g/kg and 9.45 g/kg, obvious reductions in the p-p65/p65 ratio (by 59.66% and 59.59%), p-p38/p38 ratio (by 51.67% and 72.13%), p-JNK/JNK ratio (by 55.62% and 58.80%) and p-ERKs/ERKs ratio (by 27.76% and 29.62%) were found (all *p* < 0.05 *v.s.* the IMQ group). ISDF at 3.15 g/kg only had a slight suppressive effect. The ratio of p-IKBα/IKBα was slightly reduced after treatment but without the significant differences. These results suggest that ISDF has the ability to effectively restrict the over-expression of NF-κB p65 and MAPK pathways.

### Anti-psoriatic effect of ISDF on IL-23-induced mice model

**Fig. 3:**
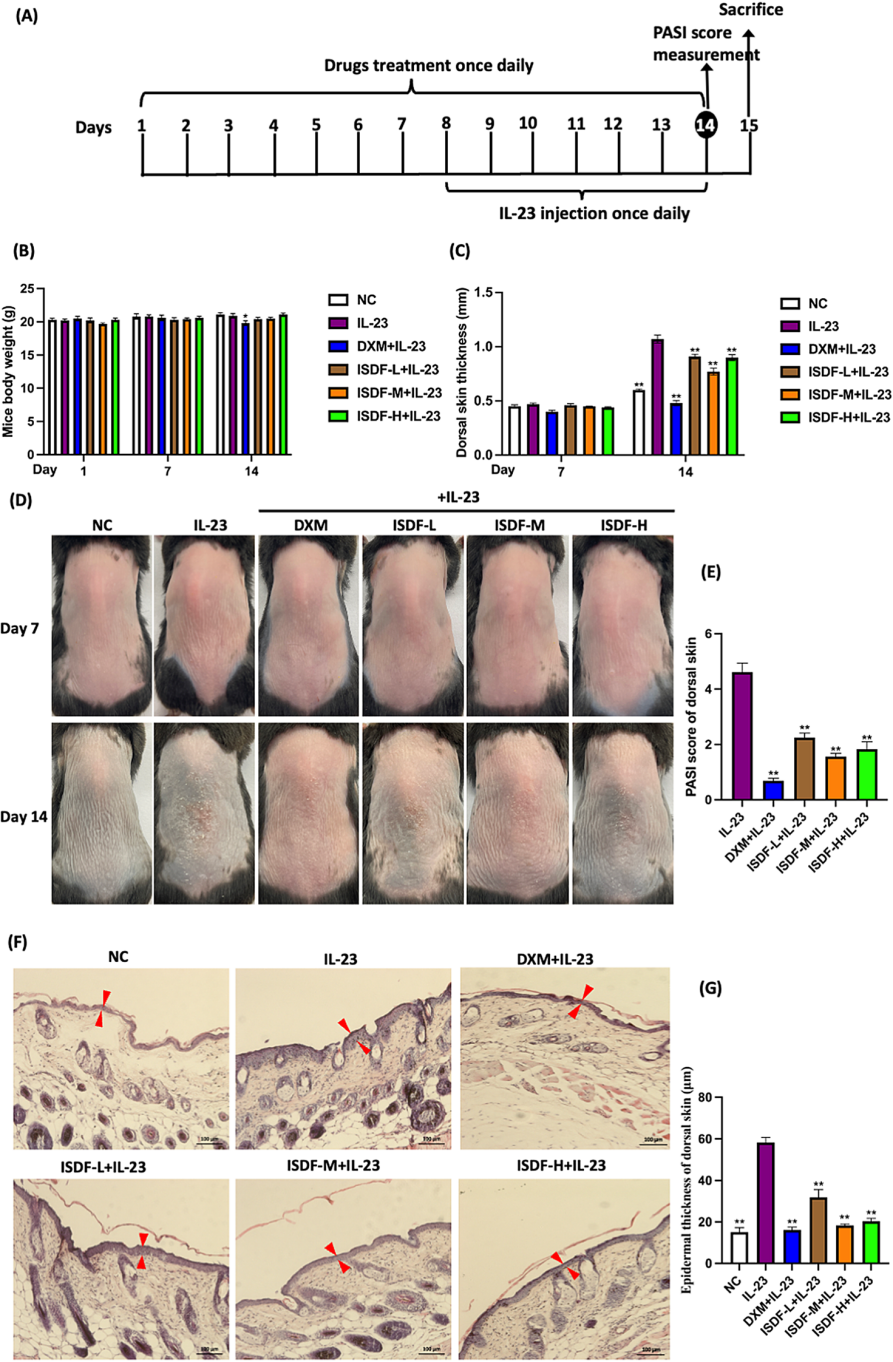
Effects of ISDF on IL-23-induced mouse psoriasis model. **A** The procedure of IL-23 inducement and ISDF treatment in mice. **B** Body weight. **C** Dorsal skin thickness. **D** Represent photos. **E** PASI score. **F** H&E-stained photos. Magnification at 100 × . **G** Epidermal thickness (indicated by arrow)

After ISDF treatment, the body weight did not change significantly in the ISDF-L/M/H groups compared to the mice in the NC group on day 14 (Fig. 3B). However, the application of DXM significantly induced a body weight reduction. This change also appeared in the IMQ-induced mice model, suggesting that DXM could cause side effects on body weight. There was no obvious body weight reduction in the IL-23-treated group or ISDF-treated groups during the experimental period. These results indicate that the application of IL-23 alone or ISDF did not exert side effects on the body weight.

After one week of IL-23 injection, the dorsal skin thickness increased by 43.92% in the IL-23 group as compared with the NC group on day 14 (Fig. 3C). After the pre-treatment of ISDF or DXM, the dorsal skin thickness of DXM-treated mice markedly decreased by 55.14%, and in the ISDF-L/M/H groups decreased by 14.95%, 28.03%, and 15.88%, respectively when compared to the IL-23 group. It shows that ISDF was effective in reducing dorsal skin thickness in mice induced by IL-23.

### Evaluation of the severity of psoriatic symptoms in the mice induced by IL-23

As depicted in Fig. 3D, while the skin lesions on the dorsal area of mice treated with IL-23 were not as intense as those observed in the IMQ-induced mice model, the characteristic symptoms such as erythema and skin scaling were visible on day 14. Following a 14-day pre-treatment with ISDF or DXM, the skin lesion severity remarkably improved, particularly in the ISDF (at the dosage of 6.30 g/kg) and DXM-treated mice. The recovery effects of ISDF (at the dosages of 3.15 g/kg and 9.45 g/kg) were also noticeable, but they were not as effective as those of ISDF (at the dosage of 6.30 g/kg) and DXM. According to the PASI score evaluated on day 14 (as shown in Fig. 3E), the scores in the ISDF-L/M/H and DXM groups were significantly reduced by 51.19%, 66.16%, 60.30%, and 85.24%, respectively, compared to the score of the IL-23 group. These results suggest that ISDF could effectively alleviate IL-23-induced psoriatic lesions in mice.

### Histopathological changes in the mice induced by IL-23

As shown in Fig. 3F, G, the epidermal thickness in the IL-23 group was markedly augmented by 2.85-fold compared to the NC group. And there was an infiltration of inflammatory cytokines in the IL-23 group. After the administration of DXM and ISDF, there was a recover in the epidermis, which thickness significantly decreased by 72.26%, 45.14%, 68.44% and 65.19% respectively in the DXM-treated and ISDF-L/M/H groups (all *p* < 0.01 *v.s.* the IL-23 group). It indicates that ISDF could effectively suppress IL-23-induced epidermal thickening.

### Biological analysis of biomarkers of mice induced by IL-23

**Fig. 4:**
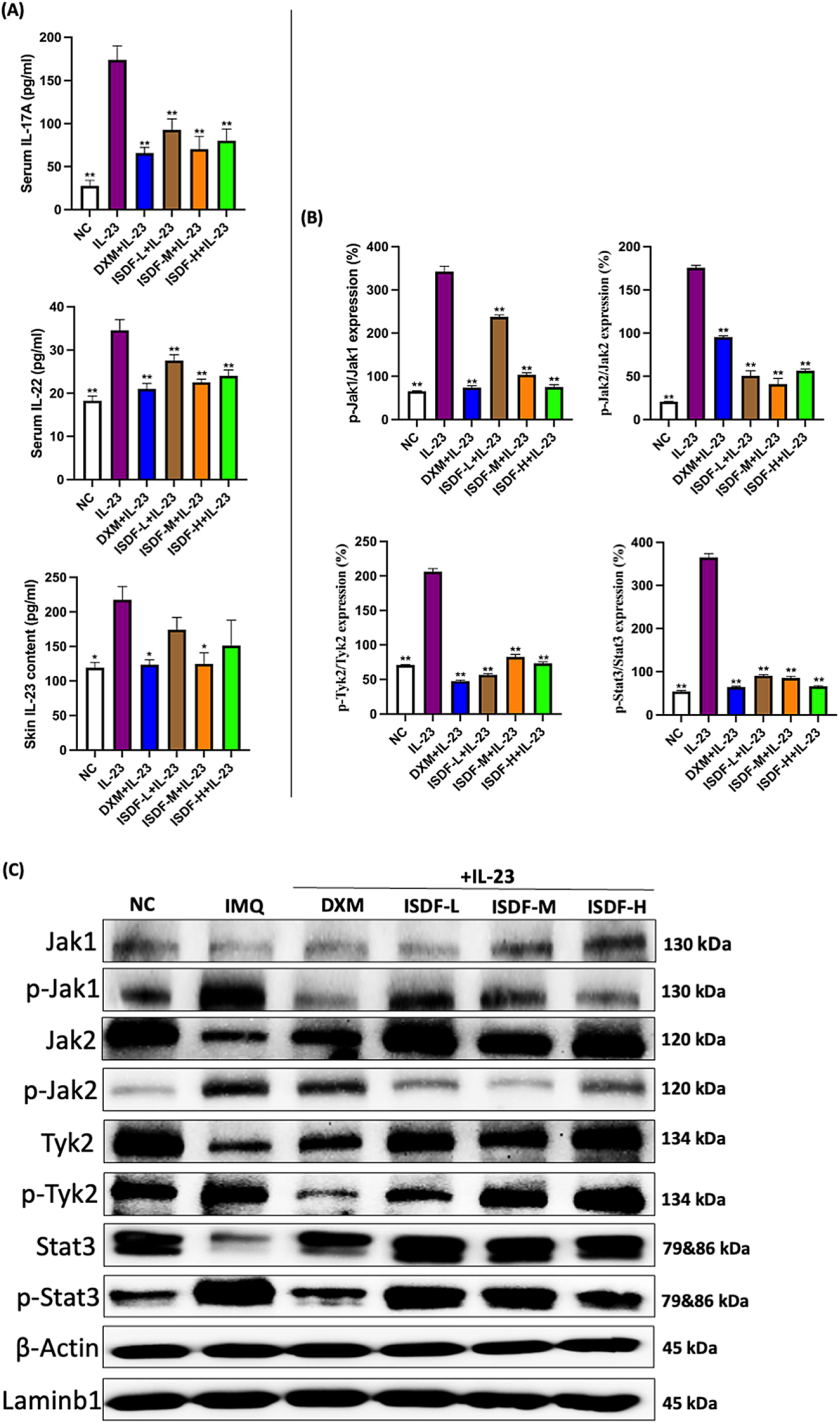
Effects of ISDF on inflammatory response induced by IL-23 in mice. **A** Serum levels of IL-17A and IL-22, and the level of IL-23 in the injected skin. **B, C** Protein expression of Jak/Stat3 pathways. **B** Results of quantitative analysis. **C** Western blotting bands. Data were presented as mean ± SEM (n = 8). *p < 0.05 and **p < 0.01 compared with the IL-23 group

Injection of IL-23 markedly increased serum IL-17A levels (by 5.26-fold) in the IL-23 group (Fig. 4A). However, the over-release of serum IL-17A was attenuated after DXM and ISDF treatment, with the reduction of 62.20%, 46.57%, 59.65%, and 53.90%, respectively, in the DXM and ISDF-L/-M/-H groups when compared to the IL-23 group. Besides, serum IL-22 was also significantly up-regulated in the IL-23 group, which was increased by 89.05% (*v.s.* the NC group) (Fig. 4A). The serum IL-22 content was significantly reduced by 39.12%, 20.28%, 34.78%, and 30.43% in the DXM and ISDF-L/-M/-H-treated groups (*v.s.* the IL-23 group). In addition, DXM and ISDF-M significantly decreased the level of IL-23 in the injected skin of IL-23-treated mice by 43.34% and 42.60%, respectively, as compared with the IL-23 group. The restrictive effect of ISDF was observed on the release of IL-23-induced IL-17A and IL-22 in the serum. The best results were obtained with ISDF administered at a dose of 6.30 g/kg, which was superior to the doses of 3.15 and 9.45 g/kg. The effectiveness of ISDF at 6.30 g/kg was comparable to that of DXM. After two weeks of treatment, ISDF was found to significantly suppress the IL-23-induced release of inflammatory cytokines.

#### Evaluation of the effect of ISDF on protein expression of Jak/Stat3 pathway in the mice induced by IL-23

The over-expressed phosphorylation of Jaks and Stat3 was stimulated after the repeated injections of IL-23, as depicted in Fig. 4B, C. The ratios of p-Jak1/Jak1, p-Jak2/Jak2, p-Tyk2/Tyk2, and p-Stat3/Stat3 were significantly increased in the IL-23-treated mice with fold changes of 4.24, 7.75, 1.91, and 5.72, respectively (*v.s.* the NC group). However, two-week ISDF treatment in advance could effectively reduce the phosphorylation of Jaks and Stat3. The levels of p-Jak1/Jak1, p-Jak2/Jak2, p-Tyk2/Tyk2, and p-Stat3/Stat3 were significantly reduced by 30.70%, 71.42%, 72.60%, and 75.34%, respectively in the ISDF-L-treated group. Similarly, these levels were decreased by 69.88%, 76.57%, 60.19%, and 76.54%, respectively in the ISDF-M-treated group, and by 78.07%, 68.09%, 64.56%, and 81.91%, respectively in the high dose of ISDF treated group (*v.s.* the IL-23 group). The study showed that ISDF, when administered at doses of 6.30 and 9.45 g/kg, had a more significant suppressive effect on the phosphorylation of p-Jak2/Jak2 than DXM. Additionally, the effect of ISDF on restricting the over-expression of p-Tyk2 and p-Stat3 was comparable to that of DXM. These findings clearly suggest that ISDF is capable of preventing the activation of Jak/Stat pathway pathways.

#### Pharmacokinetics profile of ISDF in rats

**Fig. 5:**
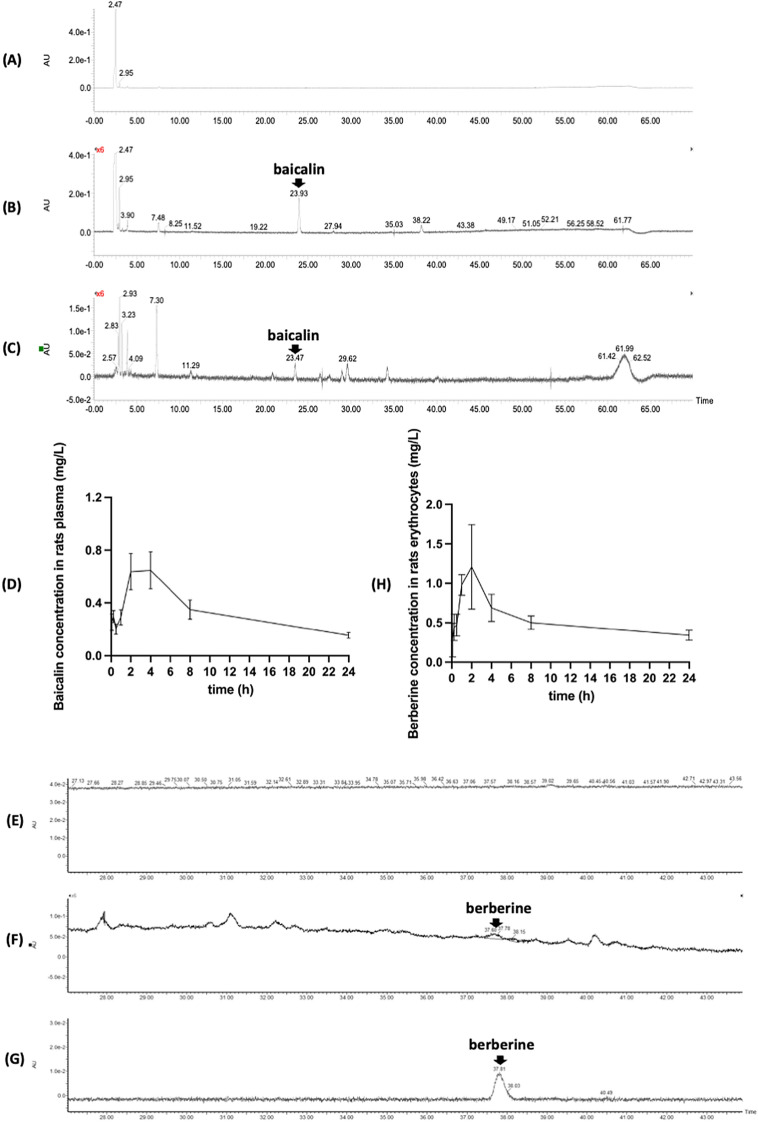
The pharmacokinetics study of ISDF in rats. **A** Chromatogram of plasma of untreated rats at 278 nm. **B** Chromatogram of baicalin standard at 278 nm. **C** Chromatogram of plasma of the ISDF-treated rats at 278 nm. **D** Plasma concentration–time curve of baicalin in the ISDF-treated rats. **E** Chromatogram of erythrocytes of untreated rats at 264 nm. **F** Chromatogram of erythrocytes of the ISDF-treated rats at 264 nm. **G** Chromatogram of berberine standard at 264 nm. **H** Erythrocytes concentration–time curve of berberine in the ISDF-treated rats

The baicalin concentration in the plasma (Fig. 5 A–C) and the berberine concentration in the erythrocytes (Fig. 5 E–G) of ISDF-treated rats were determined by HPLC analysis. The content of baicalin/berberine was calculated based on the standard curve of y = 816.16x + 472.87 (R^2^ = 0.9981)/ y = 574.3x–76.33 (R^2^ = 0.9857). A good linear relationship was obtained in the concentration range of 0.01–1.25 μg/mL (berberine) and 0.05–6.25 μg/mL (baicalin). Multi-peaks curves in concentration–time of the plasma/erythrocytes and the non-linear pharmacokinetics profile for baicalin (Fig. 3D) and berberine (Fig. 3H) were found after the oral application of ISDF in rats. As shown in Table 1, the C_max_ and T_max_ of baicalin in plasma were 0.67 ± 0.06 mg/L and 2.01 ± 0.28 h, suggesting that the absorbance of baicalin in the ISDF through the gastrointestinal tract was limited. Baicalin distribution and metabolism in ISDF-treated rats was slow, with a half-life t1/2 of 14.06 ± 3.25 h and an MRT of 7.98 ± 0.61 h. Similarly, the C_max_ and T_max_ of berberine in erythrocytes were 1.44 ± 0.71 mg/L and 2.33 ± 1.52 h, suggesting that berberine in the ISDF was mainly bound with hemoglobin and metabolised in the erythrocytes. The distribution and metabolism of berberine through erythrocytes is very slow, with a half-life t1/2 of 10.77 ± 5.80 h and MRT of 9.24 ± 1.31 h.

**Table 1:** Pharmacokinetics profile of ISDF in rats

Parameter	Unit	Value	
		Baicalin	Berberine
The maximum concentration (C_max_)	mg/L	0.67 ± 0.06	1.44 ± 0.71
Time to reach Cmax (T_max_)	h	2.01 ± 0.28	2.33 ± 1.52
Area under the curve (AUC_0-∞_)	mg/L*h	10.05 ± 0.81	24.15 ± 17.40
Mean residence time (MRT_0-t_)	h	7.98 ± 0.61	9.24 ± 1.31
The time required for drug concentration to decrease by 50% (t1/2)	h	14.06 ± 3.25	10.77 ± 5.80
The drug clearance (CL)	L/h/kg	2179.01 ± 189.41	1042.56 ± 549.50

## Discussion

In clinical practices, oral glucocorticoids and topical steroids are frequently used to quickly reduce the scales and stop inflammation in patients with psoriasis. However, their adverse effects including skin atrophy, pigmentation, and hormone-dependent dermatitis are common. Although the anti-psoriatic effects of some biologicals are very impressive, their high cost might be an unbearable burden for most patients. Since psoriasis usually runs a long course and is prone to recurrence, it is necessary to seek an alternative therapeutic approach with a lower cost, higher safety, and potent efficacy. 

Chinese herbal medicine has been applied to manage psoriasis for long. This project aimed to investigate the anti-psoriatic efficacy of an innovative Chinese herbal formula, namely ISDF, on psoriasis animal models. ISDF contains nine different Chinese herbal medicines, and most of these herbs possess strong anti-inflammatory capacities. We have reported that ISDF was capable of alleviating DNCB-induced AD-like symptoms in mice, and the mechanism was found to be closely associated with its inhibitory effect on the inflammation activated by NF-κB and MAPK pathways [13]. Since the pathogenesis of psoriasis is also centred around inflammation, we hypothesized that ISDF could also be developed into an anti-psoriasis agent. In this project, the anti-psoriatic effect of ISDF and the therapeutic target related to inflammation were evaluated using two in vivo models.

Firstly, the IMQ-induced psoriasis mouse model is generally utilized in preclinical studies because of its easy accessibility and the stability of psoriatic scales [15]. In practice, Aldara, a commercial product containing 5% IMQ (a TLR7/8 agonist), is applied to induce psoriatic skin lesions. After several applications of IMQ on the skin, characteristic psoriasis symptoms (keratinocyte hyperproliferation caused acanthosis and epidermal thickening) could be easily observed. However, the immunopathology of IMQ-induced psoriasis-like symptoms in mice mainly depends on dendritic cells and γδ T cells, a mechanism that is different from that of human psoriasis [16]. Besides, the vehicle of this commercial cream Aldara contains isostearic acid, which could also induce strong skin inflammation in a non-TLR dependent pathway [21]. Thus, the inflammation-associated gene expression of IMQ-induced mice has limited similarities with human psoriasis [22]. The IMQ-induced psoriasis model might not have the same pathogenesis as human psoriasis, but it can accurately replicate external symptoms such as silvery-white scales, dryness, itching, and red plaques. After constant daily inducement with IMQ on the dorsal area for seven days, slight skin scales could be observed and then developed into dry and flaky skin in all IMQ-treated mice on day 14. On day 21, treatment with ISDF (9.45 g/kg) or DXM for 14 days significantly suppressed the production of skin scales. During the experimental period, the mice’s body weight decreased in the DXM group, while the ISDF group remained stable. Although the dorsal skin thickness did not decrease after treatment with ISDF, which suggested that ISDF might not work through modulating the dendritic cells and γδ T cells, or could not inhibit non-TLR dependent inflammation, the PASI score showed an obvious reduction in the groups treated with ISDF on day 21. Besides, the epidermal thickness significantly decreased in the ISDF-treated groups at 6.30 and 9.45 g/kg in a dose-dependent manner. Additionally, ISDF demonstrated an inhibitory effect on the concentration of inflammatory cytokines induced by IMQ. It indicated that ISDF could work on IL-17 signaling pathways to control inflammatory conditions in psoriasis. Activation of IL-17 effectors (including IL-17A/F) triggers an inflammatory cascade which is vital in psoriasis [23], as the contents of IL-17 effectors and pro-inflammatory cytokines in psoriasis patients are usually higher than those in normal subjects [24]. Thus, biologics like bimekizumab and netakimab which target the IL-17 pathway have become the first-line strategy recently [25, 26]. According to this study, IL-17F was not found in the serum of IMQ-treated mice. However, the levels of IL-17A and IL-22 significantly reduced in ISDF-treated groups. This decrease was more pronounced at the dosage of 9.45 g/kg. The contents of serum IL-6 and skin IL-4 markedly declined in a dose-dependent manner after the administration of ISDF. It indicates that ISDF could effectively suppress the severity of psoriatic lesions induced by IMQ, the curative effect of ISDF at doses of 6.30 and 9.45 g/kg was comparable to DXM, but without adverse effects on mice body weight.

In the inflammatory cycle of psoriasis, downstream cytokine release is upregulated by IL-17 effectors of the IL-23/Th17 axis. In the IMQ-treated mice, we observed over-expression of IL-4, IL-6, IL-1β, and TNF-α. Besides, IMQ markedly up-regulated the expressions of NF-κB p-p65, p-JNKs, and MAPK p-p38. Treatment with ISDF was effective in reducing the mRNA expressions of IL-4, IL-6, IL-1β, and TNF-α, and the phosphorylation of NF-κB p65, JNKs, and MAPK p38 in mice, particularly at the middle and high doses. This suggests that ISDF can alleviate the IMQ-induced psoriatic symptoms in mice, and its therapeutic target could be the IL-23-activated Th17 signalling pathways. As a result, further research was conducted to explore the involvement of the IL-23/Th17 axis in the therapeutic effect of ISDF on psoriasis in the IL-23-induced psoriasis mouse model.

In clinical practices, the increased level of IL-23 could be observed in psoriasis patients [27]. IL-23 is essentially involved in the pathophysiology of psoriasis and acts as a principal mediator [20]. IL-23 facilitates the differentiation of Th17 cells to release mediators (IL-17A/F, IL-22, TNF-α), which leads to the over-activation of keratinocytes [8]. Therefore, IL-23 inhibitors are proven to be efficacious in managing moderate and severe psoriasis [28]. IL-23-induced psoriasis is a relevant in vivo model that can cause acute inflammation through intra-dermal injections of IL-23 [15]. In this model, IL-23 caused a psoriasis-like inflammatory condition dependent on TNF [29]. Thus, this model can mimic how the IL-23/Th17 axis works in this disease progression. However, it cannot produce all the symptoms of psoriasis, probably because only one signalling pathway is activated, and this model has not been previously used as a pre-clinical model for drug screening.

In our preliminary study, we found that IL-23-induced psoriatic scales were not as severe as those in the IMQ-induced model and could gradually recover after the last injection. To ensure that the drug treatment duration remained consistent, mice were given either ISDF or DXM for seven days prior to the first injection of IL-23. For the following seven days, the mice were given both ISDF and an injection of IL-23 simultaneously. ISDF at doses of 6.30 and 9.45 g/kg was effective in reducing psoriatic scales in mice treated with IL-23. It maintained body weight and significantly inhibited dorsal skin thickness, PASI score, and epidermal thickness. ISDF also down-regulated the over-release of IL-17A and IL-22 in the serum. It suggests that ISDF can effectively inhibit IL-23-induced inflammatory responses. To investigate the underlying mechanisms, we also assessed the effect of ISDF on the Jak/Stat3 signalling pathway. This pathway is known to activate the IL-23/Th17 axis and stimulate the keratinocytes hyperproliferation [30]. Jak inhibitors such as Tofacitinib and Deucravacitinib showed satisfactory therapeutic effects on patients with psoriasis [31, 32]. It has been observed that ISDF can efficiently inhibit the Jak/Stat3 signalling pathway by blocking the over-expression of p-Jak1, p-Jak2, p-Tyk2, and p-Stat3. It indicates that ISDF can regulate the IL-23/Th17 axis by restricting the phosphorylation of Jak/Stat3, NF-κB, and MAPK signalling pathways, which ultimately reduces the over-activation of keratinocytes and inflammation in psoriatic mice’s skin.

In our experiments, unlike the positive drug DXM, short-term treatment with ISDF (for 14 days) did not show any overt adverse effects on mice as evidenced by unchanged body weight, indicating that treatment with ISDF via gavage (up to 9.45 g/kg) was tolerable and safe for mice. The result attracted our interest in whether ISDF could be applied in long-term treatment. Therefore, we performed a pharmacokinetics study of ISDF in rats. Our previous study has determined that baicalin (about 5%) and berberine (about 3%) were two major components of ISDF [13]. After orally administering ISDF to rats, the concentration–time curve of baicalin showed only multiple peaks in the plasma. It has been recently discovered that berberine has a high rate of protein-binding, which makes it easy to bind with haemoglobin and accumulate in the blood [18, 19]. Thus, we determined the berberine concentration in the erythrocytes and also observed the multi-peaks of the concentration–time curve and the non-linear pharmacokinetics profile. Besides, the results of the pharmacokinetics study revealed that the absorption of baicalin and berberine through the gastrointestinal tract of rats was limited, and their distribution and metabolism in rats were also very slow. It prompted us to conclude that ISDF could be used in long-term application and baicalin or berberine is not the only active component of ISDF responsible for the observed therapeutic effect. We warrant further study to determine the other active compounds and verify the sub-chronic or chronic toxicity of ISDF. Other natural compounds derived from Chinese medicines such as artemisinin and quercetin which have potent anti-inflammatory activities also have great potential to be developed as anti-psoriasis agents [33]. Besides, the roles of neutrophils and dendritic cells in the psoriasis progression attract researchers’ attention. SHP2, a special phosphatase, was found to be closely related to psoriasis and its inhibitor has great potential to be novel anti-psoriasis agents [34, 35]. Investigating the effect of ISDF on the neutrophils and dendritic cells especially SHP2 could also be our next focus.

## Conclusions

**Fig. 6:**
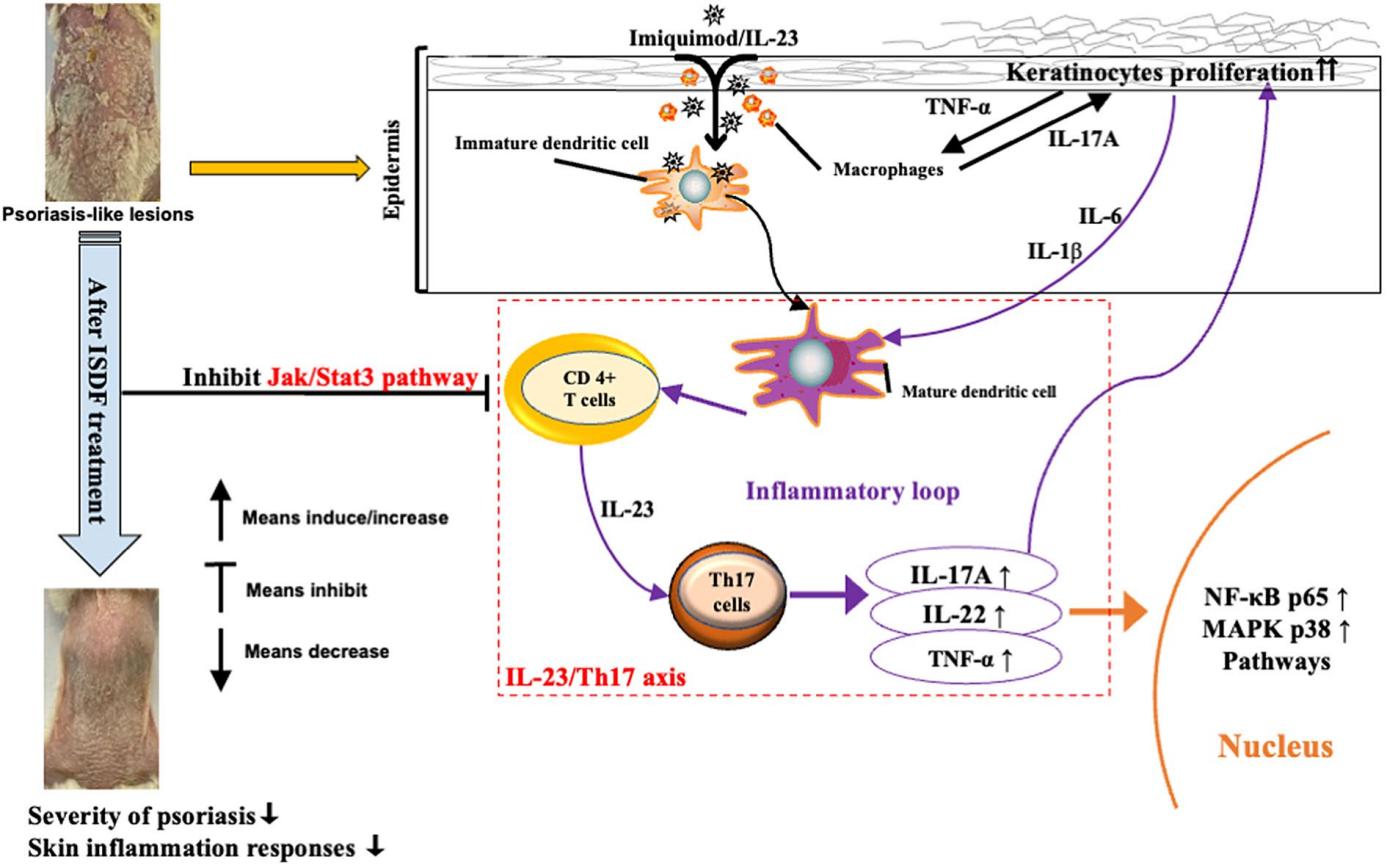
Action mechanisms of ISDF in the IMQ/IL-23-induced psoriasis mice model

This study for the first time reports the anti-psoriatic effect of an empirical Chinese herbal formula using the mice models induced by IMQ and IL-23. ISDF reduced inflammation, alleviated skin lesions, inhibited the Jak/Stat3-activated IL-23/Th17 axis, and restricted the over-expression of NF-κB and MAPK pathways (Fig. 6). ISDF has great potential to be further developed as a clinical treatment for psoriasis.

## Data Availability

The data could be provided by the corresponding author upon reasonable request.

## Abbreviations

CMC-Na: Sodium carboxymethyl cellulose
DXM: Dexamethasone
ERKs: Extracellular signal-regulated kinases
ELISA: Enzyme-linked immunosorbent assay
HPLC: High-performance liquid chromatography
ISDF: Inflammation skin disease formula
IL-23: Interleukin-23
IMQ: Imiquimod
IκBα: Nuclear factor of kappa B-cells inhibitor alpha
JNK: C-Jun N-terminal kinase
Jak: Janus kinase
Mab: Monoclonal antibody
MAPK: Mitogen-activated protein kinase
NF-κB: Nuclear factor-kappa B
PASI: Psoriasis area and severity index
Stat3: Signal transducer and activator of transcription 3
Th17: T helper 17 cells
TNF-α: Tumor necrosis factor α
